# The 30-year cardiovascular risk trajectories and their independently associated factors in participants of a Brazilian cohort (CUME Study)

**DOI:** 10.1590/0102-311XEN041323

**Published:** 2023-09-25

**Authors:** Renata Soares Passinho, Josefina Bressan, Helen Hermana Miranda Hermsdorff, Fernando Luiz Pereira de Oliveira, Adriano Marçal Pimenta

**Affiliations:** 1 Universidade Federal do Sul da Bahia, Teixeira de Freitas, Brasil.; 2 Universidade Federal de Minas Gerais, Belo Horizonte, Brasil.; 3 Departamento de Nutrição e Saúde, Universidade Federal de Viçosa, Viçosa, Brasil.; 4 Instituto de Ciências Exatas e Biológicas, Universidade Federal de Ouro Preto, Ouro Preto, Brasil.; 5 Departamento de Enfermagem, Universidade Federal do Paraná, Curitiba, Brasil.

**Keywords:** Heart Disease Risk Factors, Health Risk Behaviors, Framingham Heart Study, Cohort Studies, Fatores de Risco de Doenças Cardíacas, Comportamentos de Risco à Saúde, Framingham Heart Study, Estudos de Coortes, Factores de Riesgo de Enfermedad Cardiaca, Conductas de Riesgo para la Salud, Estudio del Corazón de Framingham, Estudios de Cohortes

## Abstract

We aimed to analyze the different trajectories of 30-year cardiovascular risk (CVR) and its independently associated factors in participants of the CUME Study, a prospective study with alumni from federal universities of Minas Gerais State, Brazil. In this study, 1,286 participants who answered the baseline (2016) and follow-up (2018 and 2020) questionnaires were included. Trajectories of CVR, according to the Framingham score, were identified with the latent class growth modelling technique with the use of the censored normal model. Analysis of the factors independently associated with each of the trajectories was conducted with multinomial logistic regression technique. Three CVR trajectories were identified: Low-Low (68.3%), Medium-Medium (26.2%), and High-High (5.5%). Male sex, living in a stable union, and having moderate and high intakes of ultra-processed foods were positively associated with the Medium-Medium and High-High CVR trajectories. Having non-healthcare professional training and working were positively associated with the Medium-Medium CVR trajectory, whereas being physically active was negatively associated with the High-High CVR trajectory. In conclusion, more than one-third of participants had CVR trajectories in the Medium-Medium and High-High categories. Food consumption and physical activity are modifiable factors that were associated with these trajectories; thus, implementing health promotion measures could help prevent the persistence or worsen of CVR. On the other hand, sociodemographic and labor characteristics are non-modifiable factors that were associated with Medium-Medium and High-High trajectories, which could help identify people who should be monitored with more caution by health services.

## Resumo

Nosso objetivo foi analisar as diferentes trajetórias de risco cardiovascular (RCV) de 30 anos e seus fatores independentemente associados em participantes do Estudo CUME, um estudo prospectivo com ex-alunos de universidades federais de Minas Gerais, Brasil. Este estudo incluiu 1.286 participantes que responderam aos questionários de linha de base (2016) e acompanhamento (2018 e 2020). As trajetórias de RCV, de acordo com o escore de Framingham, foram identificadas por modelagem de crescimento de classe latente com o uso do modelo normal censurado. A análise dos fatores independentemente associados a cada uma das trajetórias foi realizada por regressão logística multinomial. Foram identificadas três trajetórias de RCV: Baixo-Baixo (68,3%), Médio-Médio (26,2%) e Alto-Alto (5,5%). Sexo masculino, união estável e consumo moderado e alto de alimentos ultraprocessados foram positivamente associados às trajetórias de RCV Médio-Médio e Alto-Alto. Formação profissional e trabalhar em áreas não relacionadas à saúde foram positivamente associados à trajetória de RCV Médio-Médio, enquanto ser fisicamente ativo foi negativamente associado à trajetória de RCV Alto-Alto. Em conclusão, mais de um terço dos participantes apresentou trajetórias de RCV nas categorias Médio-Médio e Alto-Alto. Fatores modificáveis foram associados a essas trajetórias (consumo de alimentos e atividade física); assim, medidas de promoção da saúde podem evitar a manutenção ou a piora do RCV. Por outro lado, os fatores não modificáveis associados às trajetórias Médio-Médio e Alto-Alto (características sociodemográficas e laborais) permitem traçar o perfil das pessoas que devem ser monitoradas com mais cautela pelos serviços de saúde.

## Resumen

Nuestro objetivo fue analizar las diferentes trayectorias de riesgo cardiovascular (RCV) de 30 años y sus factores asociados de forma independiente en participantes del Estudio CUME, un estudio prospectivo con exalumnos de universidades federales de Minas Gerais, Brasil. Este estudio incluyó a 1.286 participantes que completaron los cuestionarios de referencia (2016) y de seguimiento (2018 y 2020). Las trayectorias de RCV, según el índice de Framingham, se identificaron mediante el modelado de crecimiento de clase latente utilizando el modelo normal censurado. El análisis de los factores asociados de forma independiente a cada una de las trayectorias se realizó mediante regresión logística multinomial. Se identificaron tres trayectorias de RCV: Bajo-Bajo (68,3%), Medio-Medio (26,2%) y Alto-Alto (5,5%). El género masculino, la unión estable y el consumo moderado y alto de alimentos ultraprocesados se asociaron positivamente con las trayectorias de RCV Medio-Medio y Alto-Alto. La formación profesional y el trabajo en áreas no relacionadas con la salud se asociaron positivamente con la trayectoria de RCV Medio-Medio, mientras que la actividad física se asoció negativamente con la trayectoria de RCV Alto-Alto. En conclusión, más de la tercera parte de los participantes presentó trayectorias de RCV en las categorías Medio-Medio y Alto-Alto. A estas trayectorias se asociaron factores modificables (consumo de alimentos y actividad física); por lo tanto, las medidas de promoción de la salud pueden prevenir el mantenimiento o el empeoramiento del RCV. Por otra parte, los factores no modificables asociados a las trayectorias Medio-Medio y Alto-Alto (características sociodemográficas y laborales) permiten delinear el perfil de las personas que deben ser monitoreadas con más atención por los servicios de salud.

## Introduction

Cardiovascular diseases (CVD) are responsible for high average annual direct and indirect costs worldwide ^1^ and cause significant social and economic burden, being among the main causes of death and physical disability in adults of working age ^2^. In 2020, approximately 19 million deaths were globally attributed to CVD, representing an 18.7% increase from 2010 ^1^. In Brazil, CVD also are among the main causes of death ^3^.

Moreover, in Brazil, disability-adjusted life-years (DALYs) rates for CVD standardized by age were 6,907 per 100,000 inhabitants in 1990 and 3,735 per 100,000 inhabitants in 2019. The DALYs rates for CVD were correlated with sociodemographic index of Brazilian regions, in which higher the index represents lower DALYs rates for CVD. Thus, the fastest reduction in DALYs was observed in the South and Southeast and the slowest reduction was seen in the Northeast. In 2019, 8,130,233 years of life lost (YLLs) due to CVD were recorded, affecting more individuals from 50 to 69 years of age. However, there was a reduction in the declining trend of age-standardized CVD mortality from 2016 to 2021, which highlights the need to investigate new strategies to combat this mortality, especially by implementing effective public policies ^4^.

In this context, cardiovascular risk (CVR) assessment scores can help in monitoring and decision-making since they contribute to a better diagnosis by the healthcare professional regarding the real risk of the patient developing a CVD ^5^. Moreover, the construction of CVR prediction algorithms allow the assessment of this parameter over the long term, being a useful tool in both clinical settings and public health ^6^.

The Framingham score is the most used method for long-term CVR assessment, which predicts this parameter over 10 and 30 years ^6^ ^,^ ^7^. However, no consensus is found in the literature about the relevance of estimating CVR at multiple points during an individual’s lifetime ^8^, despite some studies adopting the 30-year Framingham score for CVR estimation ^9^ ^,^ ^10^ ^,^ ^11^ ^,^ ^12^ ^,^ ^13^ ^,^ ^14^.

Long-term patterns of CVD risk scores are referred to as trajectories ^15^, and trajectory analysis in healthcare involves mapping sequences of events and various variables that contribute to health outcomes in individuals ^16^. In this context, variations in the CVR scores could occur during life, especially due to changes in lifestyle habits. Therefore, understanding how this variation occurs contributes to the planning and execution of CVD prevention programs that are more targeted to the population.

The scientific literature describes CVR trajectories according to the American College of Cardiology/American Heart Association (ACC/AHA) score ^15^; the cardiovascular health score ^17^; and the 10-year Framingham score ^18^. However, to the best of our knowledge, the 30-year Framingham score is not mentioned. Moreover, it is necessary to investigate possible changes in the trajectory of CVR and define their determinants.

The Framingham score presents modifiable components, such as body mass index (BMI), smoking, and systolic blood pressure (SBP). Increasing trends in these variables may result in long-term cardiovascular events, suggesting the need for prevention, treatment, and lifestyle modification measures ^19^.

Thus, we hypothesized that ascending and descending trajectories may occur over the 30-year CVR, along with potential determining factors that influence the modification of these trajectories.

The components of the Framingham score encompass the lifestyle habits of individuals and, consequently, variations are expected over time among the behavioral and cardiometabolic risk factors. The control of these components depends on the implementation of preventive measures, known as promoting optimal cardiovascular health. These measures include being physically active, maintaining a healthy weight, learning about cholesterol, not smoking, having a heart-healthy diet, monitoring blood pressure, having healthy sleep, and learning about blood glucose and diabetes ^20^.

Thus, this study aimed to analyze the different trajectories of 30-year CVR and their independently associated factors in participants of the Cohort of Universities of Minas Gerais (CUME Study).

## Methods

### CUME Study

The CUME Study is a prospective, open-label cohort study with a baseline data collection in 2016 and two follow-up waves in 2018 and 2020. The study participants are adults aged 18 years and older who graduated from seven federal universities in the state of Minas Gerais, Brazil. A previous publication provides detailed information about the study ^21^.

### Data collection

After signing an informed consent form, the participants completed an online questionnaire called Q_0, and the collected data composed the baseline of the cohort (2016). This questionnaire consisted of two blocks of questions. The first block included socioeconomic variables, lifestyle habits, self-reported morbidity, medication use, history of clinical and laboratory tests in the last two years, and anthropometric variables. The second block of the baseline questionnaire was presented to the participants as a validated food frequency questionnaire (FFQ), containing a set of 144 food items grouped into food groups, including dairy products, meat and fish, cereals and legumes, oils and fats, fruits and vegetables, beverages, among others ^22^.

For the two follow-up waves, the online questionnaire named Q_2 (2018) and Q_4 (2020) were completed. Q_2 covered variables about autonomy in personal hygiene, basic human needs, feeding, pregnancies since the completion of Q_0, current weight, results of recent exams, use of medications, lifestyle habits, and diagnosis of diseases during the follow-up years. Q_4 contained questions about the participant’s employment status, current weight, recent biochemical test results (triglycerides, total cholesterol, LDL cholesterol, HDL cholesterol, and blood glucose); disease diagnosis since the completion of Q_2, lifestyle habits, and sleep pattern.

### Study population

The baseline and two follow-up waves constituted the database for this study. A total of 2,499 participants completed the entire baseline questionnaire and were followed up at both two and four years. Of these, 40 foreigners, 289 Brazilians living abroad, 275 pregnant women or those within one year of giving birth, six participants with caloric intake ≤ 500kcal/day, and 208 with intake ≥ 6,000kcal/day were excluded. Thus, the initial sample included 1,681 participants.

**Figure 1:**
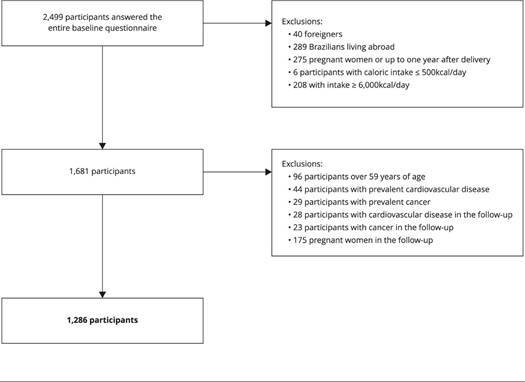
Participant inclusion flowchart in the Cohort of Universities of Minas Gerais (CUME Study), Brazil, 2016-2020.

Source: prepared by the authors.

From the 1,681 participants, those who did not meet the criteria for applying the 30-year CVR calculations ^4^ were further excluded. These exclusions consisted of participants older than 59 years (n = 96); had a prevalent CVD [cerebrovascular accident - CVA, angina, aneurysm, arrhythmia, acute myocardial infarction - AMI, heart failure, arterial insufficiency thrombosis, and those who have already undergone coronary angioplasty (n = 44)]; had prevalent cancer [lung, breast, cervical, prostate, colon, and skin cancer (n = 29)]; had CVD at the 2-year or 4-year follow-up (n = 28); had cancer at the 2-year or 4-year follow-up (n = 23); and pregnant women at the 2-year or 4-year follow-up (n = 175). Thus, the final sample size was 1,286 participants (Figure 1).

### Outcome: 30-year CVR

The 30-year CVR according to the Framingham score (coronary death, myocardial infarction, coronary heart failure, angina, ischemic, and hemorrhagic stroke, transient ischemic attack, peripheral arterial disease, and heart failure) can be calculated in two ways: using total cholesterol and HDL-c or by BMI ^6^.

In this study, the equation that measures CVR based on BMI was chosen since, in a previous study conducted with a sub-sample of the CUME Study ^23^, the self-reported data of weight, height, and BMI presented an excellent agreement with those measured directly by the researchers, presenting high intraclass correlation coefficients (ICC) of 0.989, 0.995, and 0.983, respectively. Regarding blood lipids, the self-reported data of HDL-c had a moderate agreement with those measured directly by the researchers (ICC = 0.761) and the self-reported data of total cholesterol were not validated. In that same study, the measurement of SBP (ICC = 0.667), the medical diagnosis of arterial hypertension (kappa = 0.560), and the medical diagnosis of type 2 diabetes (kappa = 0.546) were also validated ^23^.

In the baseline and follow-up questionnaires, the research participants provided the component variables of the equation that allows the calculation of 30-year CVR, being weight (kilograms) and height (meters) (BMI variable was created), sex, age, smoking status (yes or no), SBP (mmHg), use of antihypertensive medication (yes or no), and medical diagnosis of type 2 diabetes (yes or no).

### Covariates: factors associated with 30-year CVR

Covariates obtained from the baseline questionnaires were marital status, race/ethnicity, the field of study, employment status, family income, and lifestyle habits [binge drinking and physical activity], and food intake. Self-reported health status classification (good/very good, fair, and poor) was obtained by the question: “How would you classify your health status?”. Medical diagnosis of depression (yes or no) was also collected. Family income was measured in Brazilian Reais (BRL).

The alcohol consumption pattern was assessed according to binge drinking (drinking more than or equal to four doses of alcohol by women and more than or equal to five doses by men on a single occasion, considering the past 30 days) ^24^. Binge drinking was initially categorized into yes or no. Participants who answered “yes” were asked how many days of the month they were exposed to binge drinking (1 to 2 days/month, 3 to 4 days/month, and 5 or more days/month).

Physical activity was assessed by a list containing 24 leisure activities, described in minutes per week. Initially, it was categorized into light, moderate, and vigorous, and then the variable “level of physical activity” was created, categorized as “active” (≥ 150 minutes/week of moderate-intensity, ≥ 75 minutes/week of vigorous activity, or ≥ 150 minutes/week of vigorous and moderate intensity); “insufficiently active” (< 150 minutes/week of moderate-intensity, < 75 minutes/week of vigorous-intensity, or < 150 minutes/week of vigorous and moderate intensity); and inactive (absence of physical activity during leisure time) ^25^.

Information on food intake was investigated using the FFQ. Participants selected the food group items they consumed during the year before the survey and, when selecting food, they were asked to describe the size of the portions consumed in household measures (teaspoon, tablespoon, ladle, pinch, tong, saucer, cup, and glass) or traditional portions (units, slices, or pieces). Subsequently, the weekly, monthly, and annual intake frequencies of each food were transformed into daily consumption. Then, the daily food intake, in grams or milliliters, was calculated (serving size versus frequency of consumption).

The values of energy intake (kcal) and nutrients were calculated according to data provided in the *Table of Measures Referred to Foods Consumed in Brazil* ^26^, along with the *Brazilian Table of Food Composition* ^27^ and data from the United States Department of Agriculture (USDA) ^28^.

Then, the 144 food items in the FFQ were separated into groups according to the extent and purpose of industrial processing following the NOVA Classification ^29^: unprocessed/minimally processed foods, processed culinary ingredients, processed foods, and ultra-processed foods. In this study, unprocessed/minimally processed foods were grouped with processed culinary ingredients since the latter are not consumed on their own ^29^. Calorie contributions by the degree of processing were calculated from the sums of energy intakes of each food group, dividing the results by the total energy intake. These variables were divided into quintiles, with the first quintile used as the reference for data analysis.

### Statistical analysis

The 30-year CVR trajectories were identified using the group-based censored normal trajectory model. In this model, the outcome variable (cardiovascular risk) is estimated and grouped as a function of time (T0 = baseline, T2 = two years of follow-up, and T4 = four years of follow-up) by the latent class growth modelling technique ^30^.

Several models, which were distinguished according to the number of groups (from 2 to 5 groups) and shapes (linear, quadratic, and cubic), were fitted and compared using: (1) the Bayesian information criteria (BIC); (2) the mean posterior probability of assignment for each group equal to or greater than 0.7 for all groups; (3) the chance of correct classification equal to or greater than 5 for all groups; (4) the similarity between the proportion of a sample assigned to a specific group and the group probabilities estimated from the model; and (5) the narrow confidence intervals of the estimated proportion ^30^.

After the CVR trajectories were identified, the characterization of each group was performed according to the study covariates. Thus, continuous covariates were presented as means and standard deviations, and categorical covariates as relative frequencies (%).

Then, the factors independently associated with each of the CVR trajectories were investigated using the multinomial logistic regression model, with the Low-Low CVR trajectory as the reference.

Statistical significance was considered using a two-sided p < 0.05 and all analyses were performed using the software Stata, version 13 (https://www.stata.com).

This study was conducted following the guidelines established in the *Declaration of Helsinki* and all procedures involving study participants were approved by the Research Ethics Committee of the Federal University of Minas Gerais (CAAE: 44483415.5.1001.5149). Informed consent was obtained from all subjects.

## Results

### Characterization of the 30-year CVR trajectories

**Figure 2:**
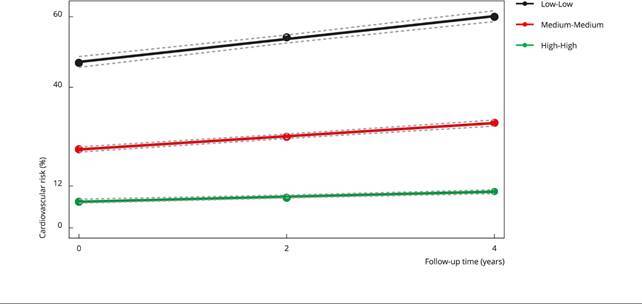
Trajectories of 30-year cardiovascular risk at four years of follow-up. Cohort of Universities of Minas Gerais (CUME Study), Brazil, 2016-2020.

Note: circles display observed values, whereas dotted gray lines represent 95% confidence intervals of adjusted trajectories. Low-Low = 68.3% of the total participants in this trajectory; Medium-Medium = 26.2% of the total participants in this trajectory; High-High = 5.5% of the total participants in this trajectory.

Figure 2 presents the 30-year CVR trajectory groups at the three analyzed times (baseline, 2-year follow-up, and 4-year follow-up) and the expected group percentages. Three distinct 30-year CVR trajectories were identified, the first with mean score values ranging from 7.5% at T0 to 10.4% at T4, the second with mean score values ranging from 22.5% at T0 to 30.1% at T4, and the third with mean scores ranging from 46.9% at T0 to 59.9% at T4. Therefore, the trajectories were named Low-Low (68.3% of the total participants), Medium-Medium (26.2% of the total participants), and High-High (5.5% of the total participants), respectively, since the mean scores varied according to the cut-off points established for the 30-year CVR categories: low risk (< 12%), intermediate risk (≥ 12% and < 40%), and high risk (≥ 40%) ^6^. Moreover, from the slope, the risks showed a slight increase for the Low-Low trajectory (2.9%), a moderate increase for the Medium-Medium trajectory (7.6%), and a high increase for the High-High trajectory (13%).

Some participant-specific changes were observed (n = 273), but they were not sufficient to change the trajectory from one 30-year CVR category to another. Of these, 38.8% (n = 106) remained in the low-risk category between the baseline and the first two years of follow-up, moving to the medium-risk category at four years of follow-up; 28.2% (n = 77) moved from the low-risk category at baseline to the medium-risk category at two years of follow-up, remaining in this last category at four years of follow-up; 14.3% (n = 39) remained in the medium-risk category between the baseline and the first two year of follow-up, moving to the high-risk category at four years of follow-up; and only 9.2% (n = 25) moved from the medium-risk category at baseline to the high-risk category at two years of follow-up, remaining in this last category at four year of follow-up. The other changes in risk categories occurred in few participants: high - medium - high (n = 2); high - medium - medium (n = 2); low - medium - low (n = 9); medium - high - medium (n = 2); medium - low - low (n = 1); medium - medium - high (n = 9); and medium - medium - low (n = 1) (data not shown).

### Participant characteristics

Demographic and socioeconomic characteristics, lifestyle habits, health conditions, and dietary intake for each of the 30-year CVR trajectories are presented in Table 1.

**Table 1:** Demographic and socioeconomic characteristics, lifestyle, health conditions, and food consumption by the 30-year cardiovascular risk score trajectory groups. Cohort of Universities of Minas Gerais (CUME Study), Brazil, 2016-2020.

Characteristics	Trajectories of 30-year cardiovascular risk (%) (n = 1,286)		
	Low-Low (n = 882)	Medium-Medium (n = 333)	High-High (n = 71)
Demographic and socioeconomic			
Gender *			
Female	77.4	41.4	33.8
Male	22.6	58.6	66.2
Age [mean (SD)] *	31.9 (5.8)	41.4 (6.8)	47.2 (6.7)
Skin color			
White	65.1	63.7	63.4
Black/Brown/Yellow/Indigenous people	34.9	36.3	36.6
Marital status *			
Without stable union	63.2	37.2	35.2
Stable union	36.9	62.8	64.8
Study area *			
Other areas	69.1	81.1	84.5
Healthcare professional	31	18.9	15.5
Level of education *			
Bachelor’s degree	29.6	19.5	23.9
Specialization degree	20.5	26.4	33.8
Master’s degree/Doctorate	49.9	54.1	42.3
Professional situation *			
Student/Retired/Housewife/Unemployed	26.4	10.5	11.3
Working	73.6	89.5	88.7
Family income (BRL) [mean (SD)] *	9,185.53 (25,608.12)	10,862.60 (6,378.58)	11,639.39 (6,914.06)
Lifestyle			
Smoking status *			
No	94.1	85.9	70.4
Yes	5.9	14.1	29.6
Binge drinking (times/month) *			
0	60.2	54.4	60.6
1-2	21.9	21.9	9.9
3-4	10.2	13.8	14.1
5 and more	7.7	9.9	15.5
Physical activity (minutes/week)			
0	21.5	25.5	31
1-149	22.3	18.9	18.3
150 and more	56.1	55.6	50.7
Food consumption (grams/day)			
Unprocessed/Minimally processed foods and processed culinary ingredients [mean (SD)]	65.9 (12.5)	65.7 (16.2)	64.4 (12.7)
Processed foods [mean (SD)] *	12.7 (6.8)	10.6 (5.4)	9.8 (5.8)
Ultra-processed foods [mean (SD)] *	22.7 (9.8)	25.3 (10.6)	26.9 (11.2)
Health conditions			
SBP (mmHg) [mean (SD)] *	113 (8.7)	121 (10.7)	167 (12.9)
Use of antihypertensive medication *			
No	99.3	88.3	50.7
Yes	0.7	11.7	49.3
Medical diagnosis of type 2 diabetes *			
No	99.7	96.7	78.9
Yes	0.3	3.3	21.1
BMI (kg/m^2^) [mean (SD)] *	23.3 (3.6)	27.1 (4.3)	30 (5.6)
Diagnosis of depression			
No	89.2	86.8	83.1
Yes	10.8	13.2	16.9
Classification of health status *			
Good/Very good	91.7	85.6	78.9
Fair	7.6	12.6	18.3
Poor	0.7	1.8	2.8

BMI: body mass index; SBP: systolic blood pressure; SD: standard deviation.

* p-value < 0.05 by Pearson’s chi-square test or analysis of variance (ANOVA) with Bonferroni correction.

The mean age of participants with Low-Low risk trajectory was 31.9 years, most being female, with high income and education, good health conditions, and healthy lifestyle habits. Participants with Medium-Medium and High-High CVR trajectories, compared with Low-Low risk participants, had higher mean age and income, and most were males, in a stable union, with non-healthcare professional training, currently working, and with worse lifestyle habits and health conditions (Table 1).

### Factors independently associated with 30-year CVR trajectories

Table 2 shows the independently associated factors with the Medium-Medium and High-High CVR trajectories, with the Low-Low risk trajectory as a reference.

**Table 2:** Independently associated factors with 30-year cardiovascular risk trajectories, using the Low-Low category as a reference. Cohort of Universities of Minas Gerais (CUME Study), Brazil, 2016-2020.

Characteristics	Trajectories of 30-year cardiovascular risk			
	Medium-Medium		High-High	
	OR	95%CI	OR	95%CI
Distal block				
Demographic and socioeconomic				
Gender				
Female	1.00 (Reference)	-	1.00 (Reference)	-
Male	4.34	3.25-5.79	5.57	3.24-9.57
Marital status				
Without stable union	1.00 (Reference)	-	1.00 (Reference)	-
Stable union	2.19	1.65-2.92	2.21	1.29-3.79
Study area				
Healthcare professional	1.00 (Reference)	-	1.00 (Reference)	-
Other areas	1.51	1.08-2.12	1.87	0.94-3.71
Working				
No	1.00 (Reference)	-	1.00 (Reference)	-
Yes	2.38	1.58-3.58	2.07	0.94-4.54
Proximal block				
Lifestyle				
Physical activity (minutes/week)				
0	1.00 (Reference)	-	1.00 (Reference)	-
1-149	0.66	0.43-1.00	0.56	0.26-1.19
150 and more	0.72	0.51-1.01	0.50	0.28-0.91
p-value for trend		0.228		0.065
Food consumption (grams/day)				
Processed food (quintiles)				
1st	1.00 (Reference)	-	1.00 (Reference)	-
2nd	1.99	1.25-3.18	1.52	0.52-4.43
3rd	2.00	1.26-3.18	3.15	1.24-8.02
4th	1.47	0.92-2.35	2.33	0.91-5.94
5th	1.52	0.95-2.44	2.76	1.10-6.88
p-value for trend		0.430		0.027
Ultra-processed foods (quintiles)				
1st	1.00 (Reference)	-	1.00 (Reference)	-
2nd	1.47	0.93-2.31	0.90	0.32-2.56
3rd	1.07	0.67-1.72	2.05	0.85-4.91
4th	1.56	0.99-2.46	2.12	0.88-5.12
5th	1.85	1.17-2.93	3.38	1.41-8.09
p-value for trend		0.036		0.003

95%CI: 95% confidence interval; OR: odds ratio.

The factors male sex, living in a stable union, having non-healthcare professional training, working, and having moderate and high consumption of processed foods and ultra-processed foods, respectively, were positively associated with the Medium-Medium CVR trajectory (Table 2).

Finally, the factors male sex, living in a stable union, having increased and high consumption of processed foods and ultra-processed foods, respectively, were positively associated with the High-High CVR trajectory. On the other hand, being physically active was negatively associated with the High-High CVR trajectory, decreasing the chances of participants belonging to this group (Table 2).

## Discussion

In this study, we identified three CVR trajectories according to the Framingham score at 30 years, denominated Low-Low (68.3%), Medium-Medium (26.2%), and High-High (5.5%) in a highly educated Brazilian population. These are similar to those found in a cohort study on risk trajectories of CVD according to the ACC/AHA risk equations ^24^: Low-Low (73.9%), Medium-Medium (21.5%), and High-High (4.6%).

This is the first study, considering an advanced search in major health databases, on CVR trajectories according to the 30-year Framingham score and its independently associated factors. Thus, comparing our scientific findings with those of other studies is challenging due to the novelty. Previous research that analyzed trajectories referred to CVR factors ^31^, lipid profile and CVD incidence ^32^, anxiety and CVD incidence ^33^, cardiovascular health and high C-reactive protein concentration ^34^, obesity and CVD risk ^35^, depression and CVD risk ^36^, and CVD risk according to ACC/AHA score and CVD incidence ^37^.

Another prospective study focusing on the early natural history of CVD since childhood, conducted with 1,288 participants and consisting of CVR trajectories mobility levels over the life course, estimated five groups of trajectories according to the 10-year Framingham score, which were different from those found in this research: High-Low (15.1%), High-High (25.7%), Medium-Low (19.4%), Low-Low (21.4%), and Low-High (18.4%) ^38^. However, the authors applied discrete mixture modelling, which is different from the latent class growth modelling technique that was used in our study.

Concerning demographic and socioeconomic characteristics, being male, living in a stable union, working, and having non-healthcare professional training care were positively associated with the Medium-Medium CVR trajectory. These same factors were positively associated with the High-High CVR trajectory, except for working and having non-healthcare professional training.

Traditional CVR factors affect both sexes however, different interactions may occur between them according to male and female coronary anatomy. The lower chances of Medium-Medium and High-High CVR trajectory in females of this study may be explained by the higher vagal tone and estrogenic levels since most participants are in the reproductive phase ^23^, which may delay the onset of coronary artery disease compared to males ^39^.

Scientific findings indicate statistically significant associations between couples and family members regarding the concordance of diagnosis of chronic noncommunicable diseases, highlighting CVD and their risk factors, especially those related to lifestyle habits ^40^. A cohort study of 236,527 Japanese couples evidenced that the risk of CVD in females whose husbands had a history of CVD may not increase. However, the risk in males whose wives had a history of CVD might increase. This difference may be explained by the dependence of males in working age on their wives concerning common lifestyle habits, particularly dietary ones ^40^.

The literature presents studies that associate exposure to long working hours. Work exposure ≥ 55 hours/week is considered sufficient evidence of harmfulness for coronary ischemic disease incidence and mortality. This relationship can be explained by the less time available to workers for activities other than work and by exposure to psychosocial, physical, biological, and behavioral risks of the work environment ^41^.

Having non-healthcare professional training was positively associated with a Medium-Medium CVR trajectory. There is evidence that associates the healthcare professionals, perceived by patients as role models regarding healthy lifestyles, with the healthier lifestyles ^42^. However, controversy exists regarding the association between lower CVR and being a healthcare professional. However, a recent systematic review has found more risk factors and worse adherence to healthy lifestyle habits among healthcare professionals, with no corresponding action between what is oriented to patients and what is practiced concerning their own health ^43^. Therefore, this scientific finding of our study should be analyzed sparingly.

Regarding the characteristics of lifestyle habits and food consumption, positive associations were found between moderate and high intakes of processed foods and the Medium-Medium and High-High CVR trajectories, respectively. Furthermore, high consumption of ultra-processed foods was also positively associated with these trajectories, whereas being physically active was negatively associated with the High-High risk trajectory.

Processed foods are industrial products made by adding salt, sugar, or other processed culinary ingredients to unprocessed or minimally processed foods. They undergo preservation methods such as canning and bottling, and examples include bread and cheese, canned vegetables, and fruit in syrup ^29^. The literature points to the harmful effects of processed meats and sodium ^44^. Furthermore, it is estimated that 10% to 53% of CVD mortality in 2048 could be prevented or delayed by reducing the intake of salt, saturated fat, trans-fats, and added sugar from lower consumption of processed culinary ingredients, processed and ultra-processed foods, and increased consumption of unprocessed/minimally processed foods in Brazil ^45^.

Biologically, the increased CVR with the consumption of ultra-processed foods [carbonated soft drinks; sweet or savory packaged snacks; chocolate, candies (confectionery); ice cream; mass-produced packaged breads and buns; margarines and other spreads; cookies (biscuits), pastries, cakes and cake mixes; breakfast “cereals”; pre-prepared pies and pasta and pizza dishes; poultry and fish “nuggets” and “sticks”, sausages, burgers, hot dogs and other reconstituted meat products; powdered and packaged “instant” soups, noodles and desserts; and many other products] still needs further scientific explanation; however, it is already known that there are mechanisms that potentiate factors related to metabolic dysfunction, inflammation, thrombus formation, oxidation, and endothelial injury ^46^. A population-based cohort study with a large sample showed that an absolute increase of 10% in the percentage of ultra-processed foods in the diet was associated with a statistically significant increase of 12%, 13%, and 11% in the rates of cardiovascular, coronary, and cerebrovascular diseases, respectively ^47^. In addition to the risk and occurrence of CVD, the consumption of ultra-processed foods is also associated with other health outcomes, such as type 2 diabetes, breast cancer, and depression ^48^.

Being physically active was negatively associated with the 30-year High-High CVR trajectory. The literature suggests that any increase in the physical activity category significantly reduces the CVR by increasing the expression of antioxidant enzymes in the heart ^49^.

### Limitations and strengths

As limitations of this study, we point to the self-reporting of the component variables of the Framingham score for estimating 30-year CVR and the factors independently associated with risk trajectories. However, we highlight that the self-reported variables that are components of the Framingham score, such as SBP, hypertension diagnosis, diabetes diagnosis, and BMI ^23^, and some variables of the factors independently associated with trajectories, particularly dietary ones ^22^, have been validated in previous studies.

Our scientific findings suggest that among working-age adults with high income and high education, the 30-year low CVR trajectory prevailed compared with those at medium and high risk. However, more than a third of the population was in the medium and high CVR trajectories, with a tendency for this outcome to worsen over time.

Male participants, who were living in a stable union and who had high intakes of processed and ultra-processed foods were more likely to have medium and high 30-year CVR trajectories. Conversely, physically active participants were less likely to have a high 30-year CVR trajectory.

Therefore, by the planning and implementation of public policies for the prevention and control of risk factors and CVD, it is essential to encourage people to adhere to healthy lifestyle habits, particularly by avoiding the consumption of processed and ultra-processed foods and engaging in regular physical activity, especially among those who have an occasional mid- or high 30-year CVR trajectory, since they tend to remain in these categories over time.

Furthermore, we highlight the feasibility of using the 30-year Framingham score in primary health care since it allows the use of non-laboratory variables, estimating CVR without greater costs for the healthcare system. In this context, we suggest implementing preventive policies and programs about health promotion and prevention of risk and cardiovascular illness.
